# Comparative Outcomes of Single- Versus Dual-Incision Approaches for Open Reduction and Internal Fixation of Complex Tibial Plateau Fractures

**DOI:** 10.3390/jcm14238281

**Published:** 2025-11-21

**Authors:** Efstratios D. Athanaselis, Theodoros Mylonas, Alexandros Koskiniotis, Alexandros A. Saridis, George Komnos, Nikolaos Stefanou, Michael Hantes, Theofilos Karachalios, Sokratis Varitimidis

**Affiliations:** Department of Orthopaedic Surgery and Musculoskeletal Trauma, University General Hospital of Larissa, 3 Panepistimiou Street, Biopolis, 41110 Larissa, Greeceakoskiniot@hotmail.com (A.K.); saridisalex@hotmail.com (A.A.S.); gekomnos@gmail.com (G.K.); hantesmi@otenet.gr (M.H.);

**Keywords:** dual-incision approach, post-traumatic osteoarthritis, Schatzker classification, single anterior incision, tibial condyles, tibial plateau fracture

## Abstract

**Background/Objectives**: Open reduction and internal fixation with plates and screws is the treatment of choice for bicondylar tibial plateau fractures. The use of a surgical approach remains a topic of debate regarding the site and number of incisions that ensure best access for reduction with minimum additional soft tissue damage. This retrospective cohort study compared clinical, radiological, and functional outcomes of single- (anterior) versus dual-incision (anterolateral and medial) approaches that are widely used in the operative treatment of Schatzker V–VI tibial plateau fractures. **Methods**: Eighty-two patients treated between 2005 and 2020 were retrospectively analyzed. Fifty-two underwent a single-incision (SI) approach and 30 a dual-incision (DI) approach. Operative parameters, complications, reduction quality, Knee Society Score (KSS), Oxford Knee Score (OKS), and post-traumatic arthritis incidence were assessed. **Results**: Mean patient age was 50.6 years, with a mean follow-up of 8.5 years. Operative time was shorter in the SI group, though fluoroscopy time was longer. No significant difference was observed in reduction quality or wound complications. Post-traumatic arthritis occurred in 57.6% of SI and 53.3% of DI patients, with severe arthritis more frequent in SI (30% vs. 12.5%, *p* < 0.05). Seven patients required conversion to total knee arthroplasty (five SI, two DI). Functional recovery was similar: mean KSS 68.6% (SI) vs. 70.5% (DI) and OKS 36.1 vs. 40.8 (*p* > 0.05) at 5 years. **Conclusions**: Both single- and dual-incision approaches for complex tibial plateau fractures provide satisfactory long-term outcomes. While differences in complications and arthritis rates were minor, surgical choice should be guided by the fracture morphology, patient characteristics, and surgeon’s experience to balance reduction quality with soft tissue preservation.

## 1. Introduction

Bicondylar tibial plateau fractures classified as types V and VI according to Schatzker are complex intra-articular fractures that disrupt the tibial joint surface and weaken the proximal tibial metaphysis bone structure. Surgical intervention is required in the vast majority of cases to restore joint congruity and stability, as well as limb axis and function [1,2,3]. According to Martz & Le Baron (2025), open reduction and internal fixation (ORIF) with plates and screws is the treatment of choice, and it should be applied as soon as the soft tissue condition allows [4,5]. The number and site of surgical approaches remain a topic of debate. Though the three-column concept and fracture fragment analysis have stressed the need for additional surgical approaches to the knee, dual- (anterolateral and medial) and single-incision (anterior) techniques are widely used for fracture fixation. Each method offers distinct advantages and drawbacks, potentially influencing both clinical and functional outcomes [6,7]. As Dobelle et al. stressed in 2024, a thorough comparison of these approaches is crucial for optimizing patient management and minimizing complications [8].

The single-incision approach, typically utilizing an anterior midline incision, aims to minimize soft tissue disruption while providing 180o access for medial and lateral plating. Proponents argue that this method reduces surgical morbidity, shortens operative time, lowers the risk of infection, and simplifies future TKA [9]. However, concerns remain regarding the adequacy of reduction, particularly for posteromedial and posterolateral fracture fragments, which may be challenging to address through a single incision.

Conversely, the dual-incision approach, incorporating usually anterolateral and medial incisions, allows for direct visualization and fixation of both tibial condyles, leading to superior biomechanical and functional outcomes in specific fracture patterns. This technique enhances fracture reduction and fixation stability but raises concerns about increased soft tissue compromise and the potential for wound complications [10].

Given the ongoing debate about these techniques, a comparative analysis of single versus dual incision for bicondylar tibial plateau fractures is expedient. This study aims to evaluate the clinical and radiological outcomes of each approach, assessing parameters such as, reduction effectiveness, complication rates, long-term functional recovery, and the incidence of post-traumatic osteoarthritis. Assuming that both methods yield comparable outcomes in terms of function and complication rates, this analysis aims to guide surgeons in selecting the most appropriate approach for the individualized operative treatment of bicondylar tibial fractures.

## 2. Materials and Methods

**Figure 1:**
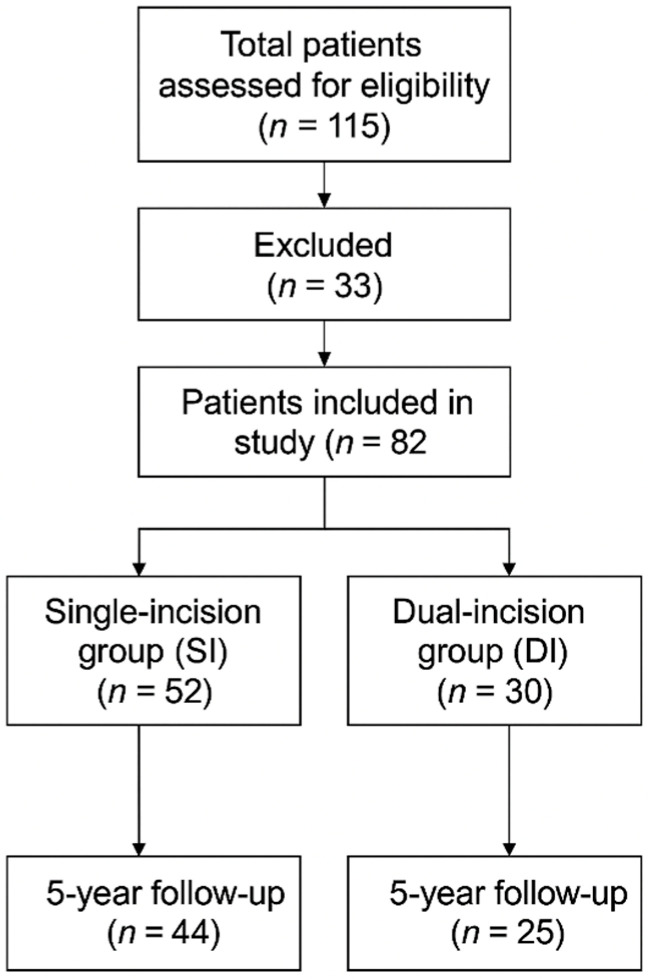
Flow chart of patients’ enrollment and allocation in the two groups.

This retrospective observational cohort study was conducted at a tertiary university hospital. Eighty-two patients who were surgically treated for bicondylar tibial plateau fractures between 2005 and 2020 were analyzed. The hospital’s surgical database and operative logs were screened for all patients treated for tibial plateau fractures between January 2005 and December 2020. Patients were categorized into two groups based on the surgical approach used: 52 patients were treated with a single-incision approach (SI group), while 30 patients underwent a dual-incision technique (DI group). Inclusion criteria consisted of skeletally mature patients with Schatzker V-VI tibial plateau fractures treated with open reduction and internal fixation (ORIF) using plates and screws. Exclusion criteria included pathological fractures, open fractures, polytrauma patients, and those with incomplete follow-up data. From an initial cohort of 115 patients with Schatzker type V–VI fractures, 33 were excluded due to loss from follow-up, open/pathological fractures, or incomplete records, leaving 82 patients for analysis (Figure 1). Ethical approval was obtained from the institutional review board (approval no. 16386/4/4/2024).

All procedures were performed in a Level I trauma center by the same surgical team using standardized techniques. In the SI group, an anterior, midline approach was utilized for reduction and fixation of both tibial condyles. In the DI group, the classic combination of an anterolateral and medial or posteromedial incision was performed to allow direct access to medial and lateral tibial plateau. The fixation method and intraoperative complications were documented. Fracture reduction was evaluated by postoperative radiological examination by two experienced orthopedic surgeons. All patients followed a standardized postoperative rehabilitation protocol, including knee immobilization in 10–15° of flexion in a brace. Range-of-motion exercises were allowed gradually after 3 weeks, and gradual weight-bearing was allowed after 6–8 weeks postoperatively.

After the early post-operative patient’s clinical evaluation, radiographic and functional outcomes were recorded annually starting at 12 months post-operatively. Long-term outcomes were assessed at the 5-year follow-up. Radiographs were independently evaluated by two blinded orthopedic surgeons, and interobserver reliability for reduction quality and arthritis grading was high (κ = 0.84). Post-traumatic arthritis was classified according to the Kellgren–Lawrence classification [11]. Reduction was classified as anatomical when the articular step was ≤2 mm, acceptable when 2–5 mm, and poor when >5 mm. Malunion was defined as >5° varus/valgus or >10° sagittal angulation. Early and late post-operative complications, including infection, wound dehiscence, and loss of reduction or implant failure, were recorded. The Knee Society Score (KSS) and the Oxford Knee Score (OKS) were used for evaluating knee function and patient satisfaction following surgical intervention.

Statistical analysis was performed using SPSS Statistics version 26.0. Continuous variables were expressed as mean ± standard deviation (SD) and compared between groups using the independent-samples *t*-test. Categorical variables were expressed as frequencies and percentages and compared using the chi-squared test or Fisher’s exact test when appropriate. Logistic regression was used to identify independent predictors of post-traumatic arthritis, adjusting for age, sex, comorbidities, fracture pattern, and reduction quality. A *p*-value < 0.05 was considered statistically significant.

## 3. Results

A total of 82 patients with bicondylar tibial plateau fractures were analyzed, with 52 operated on by a single-incision (SI group) and 30 by a dual-incision (DI group) approach. The mean patients’ age at the time of the injury was 50.6 years (36–68 years), and the mean follow-up period was 8.5 years (2.5–20 years). There was no significant difference in demographic characteristics, including age, sex, and comorbidities, between the two groups (*p* > 0.05) (Table 1).

The mean operative time was significantly shorter in the SI group compared to the DI group (70.8 min vs. 91.6 min, *p* < 0.001). The mean fluoroscopy time for validating reduction and proper plate and screw placement was longer in the SI group (mean values: 72.3 s vs. 64.1 s, *p* < 0.001). A thigh tourniquet was used in all cases, and no patient needed a blood transfusion (Table 2).

Regarding wound complications, a slightly lower incidence of wound dehiscence was marked in the SI group (11.5% vs. 16.6%, *p* = 0.53), but no case needed any further intervention. Cases of superficial infection (7.6% of the SI group and 10% of the DI group) were treated with IV antibiotics (Table 3). Elective hardware removal was carried out in 7 patients in the SI group (13.4%) and 3 in the DI group (10%) after a mean of 28 months and 29 months, respectively.

Radiographic evaluation demonstrated acceptable fracture reduction in the majority of patients in both groups. However, malunion due to malreduction or loss of reduction within the first 3–4 months postoperatively was recorded in 10 patients in the SI group (19.2%) and in 5 patients in the DI group (16.6%) by radiographic follow-up (Table 3).

Signs of mild to severe post-traumatic arthritis (stage 2–4 according to the Kellgren-Lawrence classification system) were more frequently observed during follow-up examinations in the SI group without this difference being statistically significant (57.6% vs. 53.3%, *p* > 0.05). Particularly, in the SI group, 11 patients (36.7%) had signs of stage 2 arthritis, 10 (33.3%) of stage 3, and 9 (30%) of stage 4. On the other hand, in the DI group, 10 patients (62.5%) had signs of stage 2 osteoarthritis, 4 (25%) of stage 3, and 2 (12.5%) of stage 4. Five patients in the SI group (9.6%) and 2 in the DI group (6.6%) (*p* > 0.05) underwent TKA due to symptomatic post-traumatic arthritis at a mean time of 5.5 years following the initial surgery (Table 4). The mean time to TKA did not differ significantly between groups (SI: 5.3 ± 0.75 years, DI: 5.8 ± 1.0 years; *p* = 0.52) (Table 4). Logistic regression identified a suboptimal reduction quality as an independent predictor of post-traumatic arthritis (OR 3.1, 95% CI 1.1–8.5, *p* = 0.031), while age, sex, and surgical approach were not significant predictors.

Functional outcomes were assessed at the 1 and 5 year follow-up by the Knee Society Score (KSS) and Oxford Knee Score (OKS). Comparable results between the two groups were revealed. Both scores showed satisfactory improvement in both cohorts at the first year (mean KSS: SI 74.8%, DI 76.1%, *p* = 0.38; mean OKS: SI 39.5/48, DI 40.3/48, *p* = 0.25). Eight patients from the SI group (15.4%) and five from the DI group (16.6%) were not included in the 5 year follow-up, either having already operated for TKA or being lost. In the SI group, the KSS and OKS were 68.6% and 36.1/48, respectively, whereas in the DI group, these values were 70.5% and 40.8/48 (*p* > 0.05), revealing a slight deterioration of the mean scores during the 5 year postoperative period, probably because of the evolution of post-traumatic arthritis (Table 5).

## 4. Discussion

The optimal surgical approach for type V and VI bicondylar tibial plateau fractures according to Schatzker classification remains a topic of debate, with both single- and dual-incision techniques having distinct advantages and disadvantages. Early and long-term outcomes regarding the clinical, radiographic, and functional results of our study suggest that the two approaches lead to comparable and satisfying outcomes, provided that such complex tibial plateau fractures are operated on by experienced surgeons.

**Figure 2:**
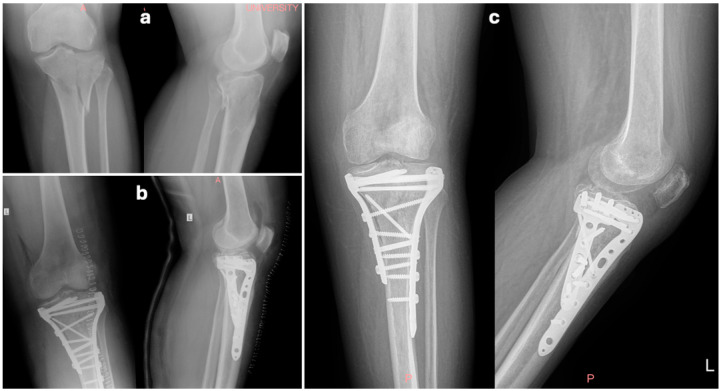
Preoperative X-rays of a 58-year-old female patient with a left Schatzker VI tibial plateau fracture (**a**). Post-operative X-rays show lateral and medial plating following reduction and grafting by an anterior midline approach (**b**). One-year follow-up X-rays (**c**) together with PROMs confirm a satisfying outcome.

**Figure 3:**
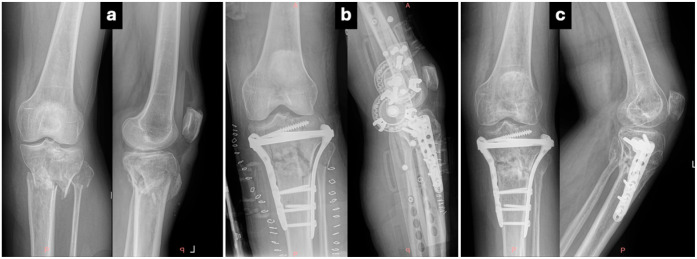
Preoperative X-rays of a 43-year-old female patient with a Schatzker VI tibial plateau fracture (**a**). Post-operative X-rays with both condyle plating (anterolateral and medial) after reduction via a dual approach (**b**). X-rays at 3-month follow-up reveal satisfying healing progress and preservation of plateau reduction despite slight anterior angulation in metaphysis, which increased the posterior slope of the tibial joint surface (**c**).

Controversy exists in the literature about the invasiveness of each method. The soft tissue envelope around the knee joint is usually injured in tibial plateau fractures, with increased severity according to fracture severity, and relative complications are not rare. Surgical approach(es) during the first 5 days’ post-traumatic pro-inflammatory phase act as additional soft tissue trauma, increasing the risk of wound healing problems, infection, and skin necrosis. Therefore, staged treatment (external fixation followed by definite osteosynthesis) is often recommended, especially in Schatzker type V–VI fractures [12,13,14]. Though alternative techniques like intramedullary nailing combined with medial plating have been proposed for minimizing surgical wounds without sacrificing biomechanical effectiveness, double plating is the gold standard for the definitive, stable fixation of complex tibial plateau fractures, and surgical approach(es) are utilized primarily or within no more than 10–15 days in case temporary external fixation has been applied [13,14,15]. Medial and lateral tibial condyle reduction and fixation demand satisfactory access, increasing the length of the anterior incision and the full-thickness skin flaps that must be created with devastating perforators in a wide area. On the other hand, considering that every surgical approach produces soft tissue trauma and compromises vascularity, it is obvious that a single incision minimizes soft tissue violation [16,17] (Figure 2 and Figure 3).

**Figure 4:**
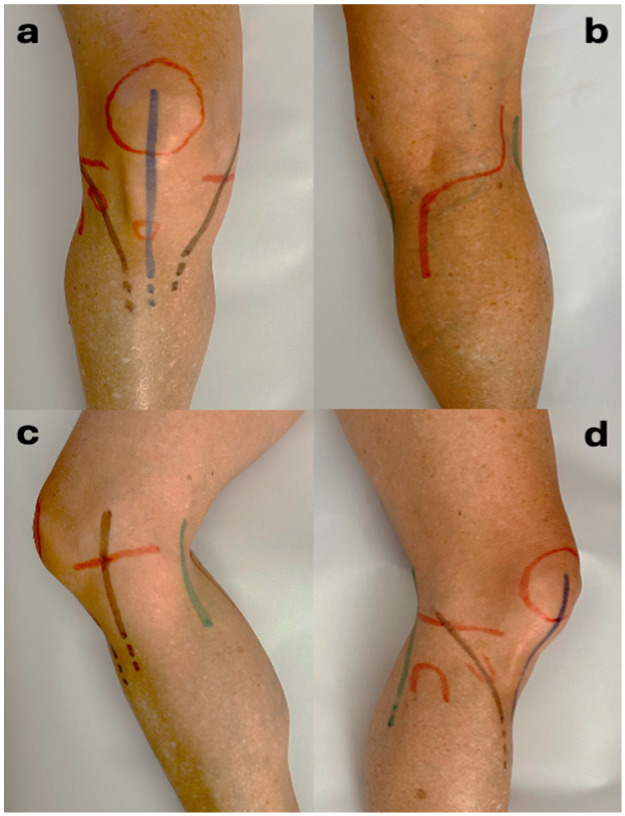
Common palpable landmarks (patella, tibial tubercle, Gerdy tubercle, fibula head, and medial and lateral joint space) are marked with red lines on right knee. Anterior (blue line), anterolateral, and medial approaches (brown lines) are drawn on anterior aspect (**a**). The posterior approach (red line), which coincides distally with the modified Lobenhoffer approach, is marked on posterior aspect (**b**). Medial (brown line) and posteromedial (green line) approaches are shown on medial aspect (**c**). Anterolateral (brown line) and posterolateral approaches (green line) are drawn on lateral aspect (**d**). It is clear that an anterior midline incision provides safety for additional approaches in case of posteromedial or posterolateral tibial plateau fragmentation.

In our study, there was no difference between the single- and dual-approach groups, regarding soft tissue complications. No patient was reoperated because of soft tissue necrosis, and only a small rate of patients (7.6% in the SI group and 10% in the DI group, *p* > 0.05) suffered from wound complications like superficial infection treated with DAIR or dehiscence (11.5% vs. 16.6%) managed conservatively without the need for reoperation. In a comparative study by Guild et al. in 2022 with 41 patients treated with single incision and 50 with dual incision, there was also no significant difference regarding postoperative soft tissue infection and necrosis rates between the two groups, though their outcomes were worse than the ones in our study (deep infection rates 22% vs. 23.5% and reoperation rates 31.7% vs. 31.4%) [18]. Moreover, additional approaches (posterolateral or posteromedial) can be safely carried out if needed, in case of a single anterior incision, while skin may be at risk due to incisions’ proximity in case the dual incision technique is used (Figure 4).

**Figure 5:**
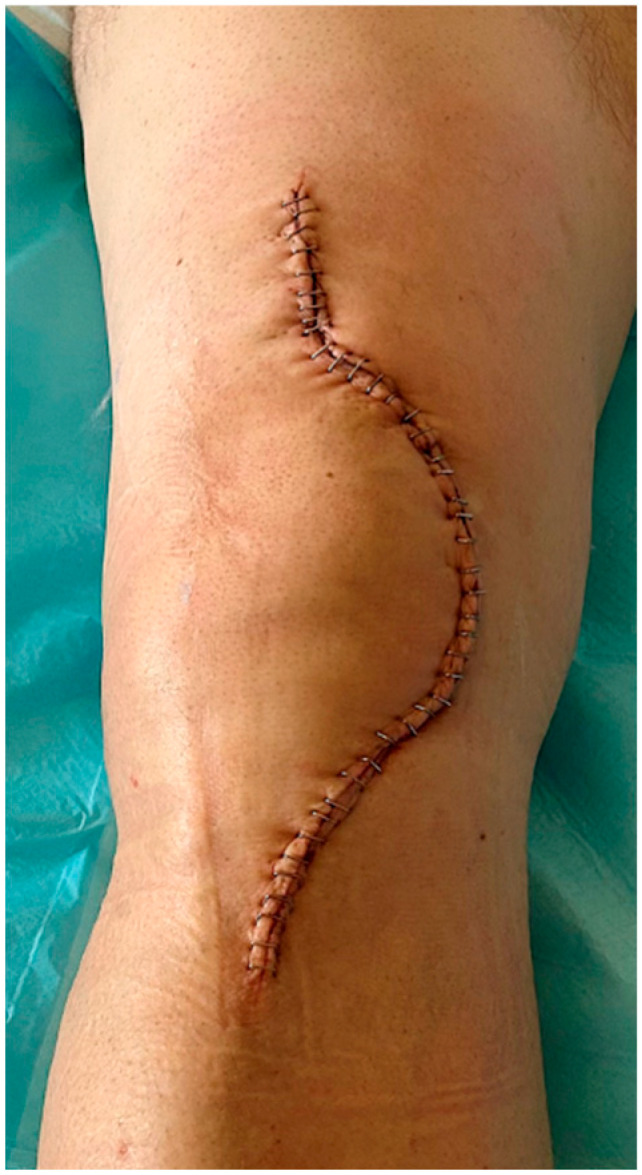
Pre-existing anterolateral approach scar implies a para-patellar incision in case of knee arthroplasty due to the risk of skin flap complications by intersecting surgical wounds.

Furthermore, anterior approach simplifies future knee reconstructive surgery. As post-traumatic knee arthritis is a quite possible long-term complication of complex tibial plateau fractures and total knee arthroplasty utilizes an anterior knee approach, the presence of two or more scars from tibial plateau fracture fixation, may jeopardize the vascularity of the skin, increasing the risk of TKA-related skin complications. Though this parameter may be underestimated by trauma surgeons, it is a major concern of those who deal with reconstructive surgery [19,20] (Figure 5).

Tibial plateau fractures, and particularly those with severe disturbance of the joint congruency, require meticulous restoration of the anatomy regarding both the tibial articular surface and the axis of the knee joint. For that purpose, adequate exposure of fracture sites and adequate access for reduction and stable fixation are prerequisites. Usually, double plating is needed for the fixation of medial and lateral tibial condyles, while free screws can be applied for specific fragment fixation and for creating a subchondral “net” to prevent joint subsidence, though biomechanically, such screws are less reliable than those locked on a plate [5]. The reported rates of inadequate fixation and malalignment show a wide range in the literature. For many authors, the dual-incision approach allows for direct visualization and fixation of both tibial condyles, supposedly leading to improved alignment and lower rates of secondary displacement, though for others, the posterolateral and/or posteromedial fragments cannot be easily fixed by traditional anterolateral and medial approaches, suggesting additional limited approaches [6,8,21]. On the other hand, the safety of anterior midline incision, particularly for fractures with less posterior displacement, has been documented [22,23]. Furthermore, no difference in the rates of malunion and malalignment was noticed postoperatively, in a study comparing a single lateral approach and plating with a dual-incision and dual plating technique in treatment of bicondylar tibial plateau fractures [24].

Though single (anterior) or dual approaches (anterolateral and medial) are widely used for ORIF, the three-column concept and fracture fragment analysis have implied the need for additional approaches [6]. Posterolateral and posteromedial fragments can be securely buttress-plated by additional modified approaches (posterolateral, posteromedial, posterior Lobenhoffer, fibular head osteotomy) [25,26] (Figure 3). However, condyle fragments inaccessible through the anterior approach can indirectly be reduced by knee joint manipulation in the varus and valgus, trans-osseous elevation through a metaphyseal window, or even percutaneously and fixed by screws applied from the anterior aspect of the tibia [12]. In our study, the dual-incision approach provided superior reduction in posteromedial fragments, but there was no statistically significant difference in the incidence of malreduction or fixation failure between the two operative techniques, indicating that a wide midline approach offers adequate access to both columns, ensuring stable fracture fixation. Moreover, no case of reoperation due to these reasons was recorded in our research data.

**Figure 6:**
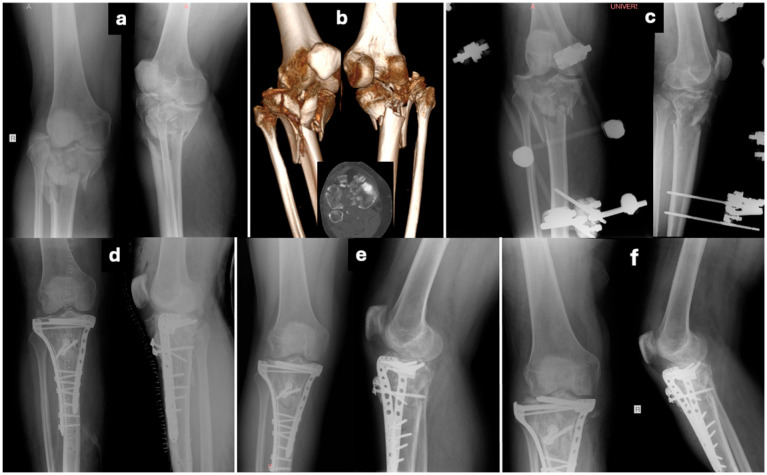
Pre-operative X-rays (**a**) of a 52-year-old male with a type-VI tibial plateau fracture according to the Schatzker classification, and anterior and posterior views of 3D reconstruction and axial CT scan (**b**). Post-operative X-rays after temporary trans-articular external fixation due to soft tissue contusion (**c**). Post-operative X-rays after ORIF of medial and lateral tibial condyle and tibial tubercle via a single anterior approach 2 weeks after injury (**d**). One-month and three-month follow-up anteroposterior and lateral X-rays, revealing progressive subsidence of the joint surface of the lateral tibial condyle (**e**,**f**).

Tibial plateau fractures are associated with post-traumatic arthritis and a long-lasting increased risk of total knee arthroplasty. Unsatisfactory intraoperative reduction is not uncommon in cases of grossly comminuted plateau fractures. Tibial joint surface subsidence, gaps, or widening of the coronal length relative to the femoral condyles are indicative of improper joint surface restoration, often leading to post-traumatic arthritis. The joint surface may also be depressed gradually postoperatively due to insufficient subchondral bone support and fixation and/or early unprotected weight bearing (Figure 6). An inadequate articular surface and lower limb axis reduction contribute to long-term joint degeneration. Various studies have stressed that even minor incongruities in the articular surface can accelerate osteoarthritis progression, potentially leading to functional limitations over time. Additionally, knee post-traumatic osteoarthritis is multifactorial, and complex tibial plateau fractures may require soft tissue repair. In fact, meniscus and ligament injuries show an increase in high-energy Schatzker V and VI tibial plateau fractures, though these are often disregarded, as bone reconstruction takes precedence [27,28,29,30,31,32]. Independent risk factors for TKA include joint depression and an increased tibial width at union, while valgus malalignment >5o is related to severe radiological osteoarthritis according to the literature, highlighting the attention that articular surface reduction and knee joint alignment require during tibial plateau fracture surgery [33,34]. However, in more recent studies, the rates of conversion to TKA are not as high as believed in the past. According to a large systematic review, the conversion rate to TKA after tibial plateau fractures is approximately 5%, without considering the type of fracture and its management [35], while in a series of 156 cases, the conversion rate to TKA was only 4%, with only 14% of the fractures being bicondylar [36]. Furthermore, surprisingly, in a series of 71 operated tibial plateau fractures at a mean follow-up of 6.17 years, Knee Injury and Osteoarthritis Outcome Score (KOOS) results revealed no correlation between anatomical/non-anatomical reduction and function, while partial articular fractures had the worst results, which are associated with the higher expectations of patients with less severe fractures [2]. However, though patients can achieve good clinical function despite radiographic findings of arthritis, in general, rates of TKA due to post-traumatic arthritis after tibial plateau fractures are not negligible. The degeneration of the operated knee joint is accelerated, patient symptoms are in relation to high-energy trauma injuries, and the incidence of post-traumatic osteoarthritis increases with higher Schatzker grades [12,30,37,38,39]. Though the rates of TKA (9.6% in SI group; 6.6% in DI group) are not significantly different from those found in the literature, the prevalence of post-traumatic arthritis evaluated according to the Kellgren–Lawrence classification system was comparable in the SI and DI groups in our study (57.6% in the SI group vs. 53.3% in the DI group), which may be explained by the severity of tibial plateau fractures included in the study (Schatzker V-VI). Effectiveness in posteromedial fragment reduction and reduced rates of post-traumatic osteoarthritis recorded in the dual-incision approach had no clinical and statistical significance. In general, comparable rates of post-traumatic knee arthritis in our study suggest that both approaches provide adequate access to fracture sites, visualization of the articular surface, and the ability to produce a stable and congruent fixation. However, the significantly higher rate of grade 4 arthritis in the SI group (30% vs. 12.5%) raises concerns over the single-incision technique regarding its reduction effectiveness, particularly in certain fracture patterns (e.g., posterior column) [40].

Limb alignment and articular surface restoration are the main goals of surgical treatment, allowing for early knee motion and rehabilitation [2,5]. Functional outcomes after tibial plateau surgical treatment depend on the surgical technique, but long-term function also depends on patient adherence to rehabilitation protocols, muscle strength, and overall joint health. While quality of fracture reduction and AO fracture classification are identified as independent predictors of clinical function, dual plating and the dual-incision technique resulted in favorable fracture reduction and satisfactory clinical outcomes, provided that minimal soft tissue disruption and proper management of osseous defects are ensured [31,41]. In another study comparing a single anterior midline incision and dual incisions in Schatzker V–VI fracture treatment, no statistically significant difference was found regarding functional outcomes, ROM, and postoperative alignment [42]. Despite the observed differences between the two groups in our study and a slight decline over the time due to post-traumatic arthritis, the functional outcomes remained comparable at short- and long-term follow-up, with both the Knee Society Score (KSS) (SI group 74.6% and 68.6% vs. DI group 76.1% and 70.5%) and Oxford Knee Score (OKS) (SI group 39.5/48 and 36.1/48 vs. DI group 40.3/48 and 40.8/48) indicating satisfying recovery in most patients, which is in agreement with favorable midterm outcomes regardless of injury severity in the literature. Of course, apart from the surgical approach, systematic rehabilitation and patient compliance with postoperative guidelines regarding mobilization, weight-bearing, and adjustments for further activities play a crucial role in optimizing outcomes [43].

To our knowledge, this is the only study evaluating the development of post-traumatic osteoarthritis in bicondylar proximal tibial fractures (Schatzker V–VI) depending on the surgical approach for dual plating. However, it has several limitations, including its retrospective design with a relatively small sample and lack of randomization, which may cause selection bias. An important weakness is our inability to assess the pre-operative arthritis of the injured knee due to the fracture disorder, which is pivotal in evaluating the post-operative deterioration of arthritis. Another limitation is the heterogeneity in follow-up duration, which limits long-term functional outcome evaluation at 5 years, though there were patients with longer follow-ups. Though the results of our study suggest comparable outcomes in agreement with other studies, further prospective randomized studies with larger sample sizes are warranted to verify our findings. Future research should also evaluate the surgical approach and fixation strategies to optimize outcomes for bicondylar tibial plateau fractures.

## 5. Conclusions

Complex Schatzker V and VI tibial plateau fracture surgery, either by the single or dual approach, is effective regarding fixation and restoration of the anatomy. In this retrospective cohort, both approaches achieved satisfactory long-term results, with slight differences in postoperative complications and post-traumatic arthritis that were not statistically significant. Surgical decision-making should be guided by individual patient characteristics, fracture morphology, and surgeon expertise to achieve satisfying reduction and mechanical stability, minimizing additional soft tissue trauma.

## Figures and Tables

**Table 1:** Demographic characteristics regarding gender, age, and follow-up period of patients included in the study.

	Single-Incision	Dual-Incision	*p*-Value
N	52	30	
male/female	36/16	18/12	0.54
Mean age (years)	51.6 ± 7 (range 39–68)	49.8 ± 6.5 (range 36–64)	0.55
Mean follow up (years)	8.9 ± 3.75 (range 3–18)	8 ± 4 (range 4–20)	0.38

**Table 2:** Intraoperative recorded parameters regarding duration of operation and image intensifier usage, as well as need for blood transfusion and type of anesthesia.

		Single-Incision (*N* = 52)	Dual-Incision (*N* = 30)	*p*-Value
Mean operative time (min)		70.8 (60–85)	91.6 (72–100)	<0.001
Mean fluoroscopic time (s)		72.3 ± 9 (55–91)	64.1 ± 12 (44–89)	<0.001
Blood transfusion (unit)		0	0	
Type of anesthesia	spinal	41 (78.8%)	22 (73.3%)	0.58
	general	11 (21.2%)	8 (26.6%)	

**Table 3:** Post-operative complications and elective hardware removal.

	Single-Incision (*N* = 52)	Dual-Incision (*N* = 30)	*p*-Value
Malunion	10 (19.2%)	5 (16.6%)	0.75
Wound dehiscence	6 (11.5%)	5 (16.6%)	0.53
Superficial wound infection	4 (7.6%)	3 (10%)	0.70
Elective hardware removal	7 (13.4%)	3 (10%)	0.76

**Table 4:** Rates of post-traumatic knee arthritis, in general, and of stages II–IV according to the Kellgren–Lawrence classification system based on radiological findings, the number of cases that ended up in total knee arthroplasty, and mean time to arthroplasty from tibial plateau fracture fixation (TKA = total knee arthroplasty).

		Single-Incision (*N* = 52)	Dual-Incision (*N* = 30)	*p*-Value
Post-traumatic arthritis		30 (57.6%)	16 (53.3%)	0.68
	mild (stage 2)	11 (36.7%)	10 (62.5%)	0.1
	moderate (stage 3)	10 (33.3%)	4 (25.0%)	0.75
	severe (stage 4)	9 (30.0%)	2 (12.5%)	0.25
Post-traumatic TKA (%)		5 (9.6%)	2 (6.6%)	0.72
Mean time to TKA (years)		5.3 ± 0.75 (4–7)	5.8 ± 1 (4–8)	0.52

**Table 5:** Functional outcome evaluated by Knee Society Score and Oxford Knee Score at 1 and 5 year follow-up for single- and dual-incision group.

	Follow-Up	Single-Incision		Dual-Incision		*p*-Value
Knee Society Score (KSS)	1 year	*N* = 52	74.8% (59–85) (S.D. = 6.9)	*N* = 30	76.1% (61–84) (S.D. = 6)	>0.05
	5 years	*N* = 44	68.6% (52–84) (S.D. = 7)	*N* = 25	70.5% (50–87) (S.D. = 6.4)	
Oxford Knee Score (OKS)	1 year	*N* = 52	39.5/48 (32–43) (S.D. = 2.3)	*N* = 30	40.3/48 (34–44) (S.D. = 2.75)	>0.05
	5 years	*N* = 44	36.1/48 (28–44) (S.D. = 2.6)	*N* = 25	38.8/48 (30–44) (S.D. = 2.9)	

## Data Availability

The data presented in this study are available on request from the corresponding author.

## Abbreviations

The following abbreviations are used in this manuscript:

SI	Single Incision
DI	Dual Incision
KSS	Knee Society Score
OKS	Oxford Knee Score
ORIF	Open Reduction Internal Fixation
TKA	Total Knee Arthroplasty
IV	Intravenous
SD	Standard Deviation
